# Enhanced Activity of Exportin-1/CRM1 in Neurons Contributes to Autophagy Dysfunction and Senescent Features in Old Mouse Brain

**DOI:** 10.1155/2021/6682336

**Published:** 2021-08-13

**Authors:** Elisa Gorostieta-Salas, Daniel Moreno-Blas, Cristian Gerónimo-Olvera, Bulmaro Cisneros, Felipe A. Court, Susana Castro-Obregón

**Affiliations:** ^1^Departamento de Neurodesarrollo y Fisiología, División de Neurociencias, Instituto de Fisiología Celular, Universidad Nacional Autónoma de México (UNAM), Mexico City, Mexico; ^2^Center for Integrative Biology, Faculty of Sciences, Universidad Mayor, Santiago, Chile; ^3^Department of Genetics and Molecular Biology, Center of Research and Advanced Studies (CINVESTAV-IPN), Mexico City, Mexico; ^4^Fondap Geroscience Center for Brain in Health and Metabolism, Santiago, Chile; ^5^Buck Institute for Research on Aging, Novato, USA

## Abstract

Brain aging is characterized by dysfunctional autophagy and cellular senescence, among other features. While autophagy can either promote or suppress cellular senescence in proliferating cells, in postmitotic cells, such as neurons, autophagy impairment promotes cellular senescence. CRM1 (exportin-1/XPO1) exports hundreds of nuclear proteins into the cytoplasm, including the transcription factors TFEB (the main inducer of autophagy and lysosomal biogenesis genes) and STAT3, another autophagy modulator. It appears that CRM1 is a modulator of aging-associated senescence and autophagy, because pharmacological inhibition of CRM1 improved autophagic degradation in flies, by increasing nuclear TFEB levels, and because enhanced CRM1 activity is mechanistically linked to senescence in fibroblasts from Hutchinson–Gilford progeria syndrome patients and old healthy individuals; furthermore, the exogenous overexpression of CRM1 induced senescence in normal fibroblasts. In this work, we tested the hypothesis that impaired autophagic flux during brain aging occurs due to CRM1 accumulation in the brain. We found that CRM1 levels and activity increased in the hippocampus and cortex during physiological aging, which resulted in a decrease of nuclear TFEB and STAT3. Consistent with an autophagic flux impairment, we observed accumulation of the autophagic receptor p62/SQSTM1 in neurons of old mice, which correlated with increased neuronal senescence. Using an in vitro model of neuronal senescence, we demonstrate that CRM1 inhibition improved autophagy flux and reduced SA-*β*-gal activity by restoring TFEB nuclear localization. Collectively, our data suggest that enhanced CRM1-mediated export of proteins during brain aging perturbs neuronal homeostasis, contributing to autophagy impairment, and neuronal senescence.

## 1. Introduction

Aging is defined as a time-dependent functional decline that increases the likelihood to die [1], although it affects different species at variable rates [2, 3]. The aging process involves the combined action of a set of different molecular and cellular mechanisms including genomic instability, mitochondrial dysfunction, proteostasis failure, and cellular senescence, collectively coined the hallmarks of aging [4]. Dysfunctional autophagy is associated with numerous age-related diseases including metabolic and neurodegenerative disorders [5]. Particularly in the brain, autophagy failure and the accumulation of senescent cells are strongly associated with brain deterioration and lose of cognitive abilities, both in physiological and pathological aging [6, 7].

Macroautophagy (referred to as autophagy) is a catabolic process whereby cytoplasmic components and damaged organelles like mitochondria and misfolded proteins are engulfed in a double membrane vesicle called autophagosome and transported then to lysosomes for degradation [8]. Since autophagy is involved in the regulation of different cellular process including protein turnover and energy metabolism, it is considered as a quality control mechanism of the cell [9]. Increasing evidence indicates that a deficient autophagic degradation promotes cellular senescence in proliferating and postmitotic cells [10–13].

Senescent cells are characterized by a lack of response to mitotic and apoptotic stimuli, leading to a perdurable cell cycle arrest accompanied by the expression of the tumor suppressors *Cdkn1a* (coding for p21^CIP/WAF^) and/or *Cdkn2a* (coding for p16^INK4A^) and antiapoptotic *Bcl2* family members, as well as activation of the lysosomal enzyme senescence-associated *β*-galactosidase (SA-*β*-gal) [14]. The most important feature of cellular senescence is the acquisition of a senescence-associated secretory phenotype (SASP) which depending on the physiological context can be either beneficial or harmful. In early stages of senescence, cells secrete growth factors and proteases that facilitate tissue repair and remodeling, as well as cytokines that promote infiltration of effector immune cells. Yet, persisting signaling is tumorigenic and causes chronic inflammation, a major contributor to age-related dysfunction. SASP molecules also have an autocrine role that enhances the senescent phenotype and a paracrine role that induces senescence in surrounding cells, altering thereby the tissue where they reside [15]. Therefore, it is fundamental to understand the molecular basis underlying the establishment and maintenance of senescence. Senescent cells have an aberrant nuclear morphology and alterations of proteins that conform the nuclear envelope, which contribute, at least in part, to trigger cellular senescence. For example, lamin A/C stabilization leads to the degradation of polycomb repressive complex 1 (PCR1), which promotes the *Cdkn2a* expression [16], while loss of lamin B1 and its receptor LBR1 induces senescence [17]. LBR1 reduction causes changes in the chromatin structure that upregulate in turn the expression of SASP factors, such as IL-6, IL-8, and MMP1 [18]. Relevant to brain aging, treatments with rapamycin or trehalose that restore dysfunctional autophagy in long-term cultured neurons ameliorate senescent features, including SA-*β*-gal activity, nuclear envelope alterations, and p16^INK4A^ expression [13, 19, 20].

A previously unrecognized feature of aging is an enhanced CRM1 (exportin-1/XPO1)-driven nuclear protein export mechanism [21], which was recently observed in skin fibroblasts from both Hutchinson–Gilford progeria syndrome (HGPS) patients and old healthy individuals [21]. It is though that enhanced nuclear protein export, due to increased activity of CRM1, impacts protein homeostasis by altering nucleocytoplasmic partitioning of critical proteins (transcription factors, enzymes, and structural proteins). Supporting this hypothesis, pharmacological attenuation of CRM1 activity in HGPS fibroblast was sufficient to prevent premature senescence, while the CRM1 overexpression in normal human skin fibroblasts induces them to senesce [21]. CRM1 binds to cargo proteins through the recognition of a hydrophobic nuclear export sequence (NES) to regulate the nucleocytoplasmic transport of hundreds of proteins [22], including the transcription factor EB (TFEB), the main inducer of autophagy, and lysosomal biogenesis gene expression [23, 24]. In line with this, reduction of the CRM1 expression promotes autophagy, via TFEB nuclear enrichment, which ultimately extends lifespan in worms and protects from neurodegeneration in flies [24]. STAT3, other regulator of autophagy, is also a CRM1 target. Cytoplasmic STAT3 suppresses autophagy by binding to PKR, which interferes then with eIF2*α* phosphorylation, a necessary step for autophagy induction [25].

Previously, we described that autophagy dysfunction causes neuronal senescence [13]; however, the molecular mechanisms underlying autophagy dysfunction during aging and the consequent induction of cellular senescence are still poorly understood. In this study, we explored the idea that the age-associated perturbation of the autophagic flux, with the concomitant induction of neuronal senescence, might be mechanistically linked to the increased expression and activity of CRM1 that could occur during aging. We found indeed that the CRM1 protein level and activity increased in the mouse hippocampus and cortex during physiological aging, provoking then a decrease of nuclear TFEB and STAT3. The physiological consequence of this alteration is an impaired autophagic flux, as shown by accumulation of p62/SQSTM1 in neurons of old mice and increased neuronal senescence. Remarkably, CRM1 inhibition increased nuclear TFEB and restored thereby the autophagic flux in cultured neurons, as demonstrated by reduced p62/SQSTM1 and LC3 accumulation, increased functional lysosomes, and reduced SA-*β*-gal activity. Taken together, our data imply that enhanced CRM1-mediated export of proteins during brain aging perturbs neuronal homeostasis, contributing to autophagy impairment and potentially to neuronal senescence.

## 2. Materials and Methods

### 2.1. Animals

Wild-type (WT) C57BL/6J [International Mouse Strain Resource (IMSR) catalog #JAX: 000664, RRID: IMSR_JAX: 000664] was obtained from Jackson Laboratory and maintained in the Universidad Mayor animal facility. Autophagy reporter GFP-LC3 transgenic mice C57BL/6 J were kindly shared by Dr. Sandra Cabrera and kept at the animal house of the Institute of Cellular Physiology at the National University of Mexico (UNAM), housed at 22°C in 12 h light/12 h dark cycle with ad libitum access to water and food. Mice used in the present study were handled and cared according to the animal care and ethics legislation. All procedures were approved by the Internal Committee of Care and Use of Laboratory Animals of the Institute (IFC-SCO51-18) and the SAG Chile (RUP: 13.1.07.0018).

### 2.2. Cortical Primary Cultures and Leptomycin B Treatment

Cortical primary cultures were prepared as previously described (Moreno-Blas et al. 2019). Cerebral cortices from Wistar rat embryos of 17 days of gestation were isolated and dissociated by digestion and trituration with a Pasteur pipette in a solution of 1 : 1400 Trypsin-EDTA (15400054, Invitrogen/Gibco, Grand Island, NY, USA). Cells were suspended in neurobasal medium (21103049, Invitrogen/Gibco, Grand Island, NY, USA) supplemented with 2% B27 (17504044, Invitrogen/Gibco, Grand Island, NY, USA), 200 mM GlutaMAX™ Supplement (35050061, GIBCO Life Technologies, Grand Island, NY, USA), and 0.02 mg/ml gentamicin (15710064, Invitrogen/Gibco, Grand Island, NY, USA). Cells were plated at a density of 1.05 × 10^5^/cm^2^ in 12-well plates precoated with poly-L-lysine (P1524, Sigma-Aldrich St. Louis, MO, USA) (0.01 mg/ml). Cultures were maintained up to 26 days in vitro (26 DIV) at 37°C in a humidified, 5% CO_2_ atmosphere. Half of the medium was changed every 6 days. Cortical cells were treated with 5 nM leptomycin B (LMB; Sigma-Aldrich) for 24 h (at 25 DIV) or 4 days (at 22 DIV) diluted in ethanol (vehicle) and 20 *μ*M chloroquine (C-6628 Sigma-Aldrich , St. Louis, MO, USA) for 4 h.

### 2.3. Immunoblotting Analysis

#### 2.3.1. Brain

Animals were euthanized via an overdose of pentobarbital. Hippocampus and cerebral cortex from six young (3 to 6 months), six adult (14 to 17 months), and three-old (24 to 28 months) wild-type mice were homogenized in an extraction buffer containing (Tris-HCl pH 8.0 50 mM, NaCl 150 mM, Triton X-100 1%, sodium deoxycholate 0.5% and SDS 2%). Thirty micrograms of total protein was separated by SDS-PAGE and electroblotted onto polyvinylidene (PVDF) membranes (Millipore). Membrane was blocked with 3% Blotting-Grade blocker (BIO-RAD Cat. #170-6404) and incubated overnight with the primary antibody at 4°C, rabbit anti-CRM1 (1 : 1000, Novus 100-79802, Centennial-CO, USA), rabbit anti-lamin B1 (1 : 1000, Abcam 32454, Cambridge, MA, USA), mouse anti-lamin A/C (1 : 1000, Santa Cruz Biotechnology 376248, Dallas, TX, USA), and mouse anti-*β*-actin (1 : 5000, Santa Cruz Biotechnology 47778, Dallas, TX, USA), diluted in TTBS/BSA 3%. After three washes with TTBS, secondary antibody goat anti-mouse IgG (1 : 5000, Sigma A3682, Saint, Louis, USA) or goat anti-rabbit IgG (1 : 5000, Sigma A0545, Saint, Louis, USA) was prepared in TTB/blocker 3%. Following by three washes, immunoreactivity was detected by chemiluminescent HRP substrate (Milipore Cat. WBKLS0100, Billerica, Ma, USA). Membranes were scanned using C-DiGit Blot Scanner (LI-COR).

For subcellular fractions, the collected tissue was homogenized using a hypotonic buffer (Hepes 10 mM, MgCl_2_ 1.5 mM, KCl 10 Mm, pH 7.4), incubated for 5 min at 4°C and centrifuged at 11,000 rpm for 1 min at 4°C. To collect cytoplasmic proteins, supernatant was collected and centrifuged again at 4,000 rpm for 10 min at 4°C. The supernatant was collected again; to precipitate cytoplasmic proteins, a 1 : 1 proportion of methanol/chloroform mix (5 : 1) was added and centrifuged at 14,000 rpm for 5 min. The pellet obtained was resuspended in lysis buffer (Tris-HCl pH 8.0 50 mM, NaCl 150 mM, Triton X-100 1%, sodium deoxycholate 0.5%, and SDS 2%). To collect nuclear proteins, the pellet of the first centrifugation was resuspended in hypotonic buffer and centrifuged at 4,000 rpm for 5 min at 4°C. Supernatant was discarded, and the pellet was resuspended on lysis buffer.

Thirty micrograms of cytosolic and nuclear fractions was separated by SDS-PAGE and electroblotted onto polyvinylidene (PVDF) membranes (Millipore). Membranes were blocked with 3% Blotting-Grade blocker (BIO-RAD Cat. #170-6404) and incubated overnight with the primary antibody at 4°C, anti-TFEB (1 : 1000, Mybiosure MBS716265, San Diego, CA, USA), anti-GADPH (1 : 8000, Cell Signaling Technology Gapdh 14c10 anti-rb mAb 2118), and rabbit anti-fibrillarin (1 : 1000, Abcam 5821 Cambridge, MA, USA), diluted in TTBS/BSA 3%. Secondary antibody goat anti-mouse IgG (1 : 5000, Sigma A3682, Saint, Louis, USA) or goat anti-rabbit IgG (1 : 5000, Sigma A0545, Saint, Louis, USA) was prepared in TTB/blocker 3%. Immunoreactivity was detected by chemiluminescent HRP substrate (Milipore Cat. WBKLS0100, Billerica, Ma, USA). Membranes were scanned using C-DiGit Blot Scanner (LI-COR).

#### 2.3.2. Primary Culture

Cells were lysed in an extraction buffer consisting of 62.5 mM Tris, 2% SDS, and 2 mg/ml protease and phosphatase inhibitors 18 (Complete, Roche Molecular Diagnostics, pH 7.4). Thirty micrograms of total protein was separated by SDS-PAGE and electroblotted onto polyvinylidene fluoride (PVDF-FL) membranes (Millipore). Membranes were incubated overnight with the primary antibody at 4°C, rabbit anti-LC3 (1 : 2000, MBL PD014, Nagoya, Japan), rabbit anti-lamin B1 (1 : 1000, Abcam 32454, Cambridge, MA, USA), mouse anti-lamin A/C (1 : 1000, Santa Cruz Biotechnology 376248, Dallas, TX, USA), and mouse anti-*β*-actin (1 : 10000, Santa Cruz Biotechnology 47778, Dallas, TX, USA) diluted in TTBS/BSA 3%. Following three washes with TTBS secondary antibody IRDye® 680RD goat anti-rabbit (925-68071, LI-COR) or IRDye® 800CW goat anti-mouse (925-32210, LI-COR) that was applied at 1 : 5000 dilution in TTBS, membranes were scanned and analyzed using an Odyssey® IR scanner and Odyssey® Image Studio software 5.2.5.

### 2.4. Immunofluorescence

#### 2.4.1. Brain Section

GFP-LC3 transgenic mice under anesthesia with pentobarbital sodium were perfused transcardially with PBS 1×, then with 4% paraformaldehyde. Brains were drop fixed in 4% paraformaldehyde for 24 h; cryoprotection brains were immersed in PBS/30% sucrose for 24 h. Brain coronal sections (40 *μ*m) from the hippocampus and perirhinal cortex were mounted serially. The sections were permeabilized and blocked with PBS 1×/0.5% Tween/BSA 5% for 30 min at RT. After three washes, primary antibody was incubated at 4°C overnight in PBS/0.05% Tween/0.1% Triton. Next, sections were incubated with Alexa Fluor-conjugated secondary antibodies (1 : 400, Life Technologies, Oregon, USA) in PBS/0.05% Tween/0.1% Triton for 12 h at 4°C; nuclei were stained with DAPI (1 *μ*g/ml). To avoid lipofuscin, autofluorescence slices were incubated with Sudan Black B. The following primary antibodies were used: rabbit anti-CRM1 (1 : 500, Novus NB100-79802, Centennial-CO, USA), rabbit anti-TFEB (1: 400, Mybiosure MBS716265, San Diego, CA, USA), mouse anti-p62 (1 : 300, Abcam 56416, Cambridge, MA, USA), mouse anti-lamin A/C (1 : 1000, Santa Cruz Biotechnology 376248, Dallas, TX, USA), rabbit anti-p21 (1 : 200, abcam 109199 Cambridge, MA, USA), mouse anti-p16 (1 : 200, Santa Cruz Biotechnology sc1661, Dallas, TX, USA), mouse anti-class III *β*-tubulin (1 : 1000, Abcam 14545, Cambridge, MA, USA), rabbit anti-class III *β*-tubulin (1 : 1000, abcam 18207, Cambridge, MA, USA), and rabbit anti-MAP2 (1 : 500 abcam 32454 Cambridge, MA, USA), mouse anti-GFAP (1 : 400, Sigma-Aldrich G3893, St. Louis, MO, USA), and mouse anti-STAT3 (1 : 100 Santa Cruz Biotechnology 482, Dallas, TX, USA). Images were acquired using a confocal microscope Zeiss LSM 800; in all cases, images were processed and quantified using Fiji software. Pseudocolors were assigned to gray images. Corrected fluorescence intensity was obtained as the result of raw fluorescence intensity minus background fluorescence in every case.

#### 2.4.2. Primary Culture

Cells were fixed with 100% methanol on ice for 20 min and then rinsed with PBS blocked with PBS/5% BSA, and incubated at 4°C with primary antibody overnight. Alexa Fluor-conjugated secondary antibodies were diluted in PBS/2% BSA (1 : 500, LIFE TECHNOLOGIES, Oregon, USA) and incubated for 1 h at room temperature. Nuclei were stained for 5 min with DAPI (1 *μ*g/ml). The antibodies used and dilutions were as follows: rabbit anti-CRM1 (1 : 400, Novus NB100-79802, Centennial-CO, USA), rabbit anti-TFEB (1: 500, Mybiosure MBS716265, San Diego, CA, USA), mouse anti-p62 (1 : 500, Abcam 56416, Cambridge, MA, USA), rabbit anti-LAMP1 (1 : 1000, Sigma-Aldrich L1418, St. Louis, MO, USA), mouse anti-lamin A/C (1 : 1000, Santa Cruz Biotechnology 376248, Dallas, TX, USA), rabbit anti-lamin B1 (1 : 1000, Abcam 32454, Cambridge, MA, USA), mouse anti-p16 (1 : 1000, Santa Cruz Biotechnology sc1661, Dallas, TX, USA), mouse anti-class III *β*-tubulin (1 : 1000, Abcam 14545, Cambridge, MA, USA) and rabbit anti-class III *β*-tubulin (1 : 1000, abcam 18207, Cambridge, MA, USA). Images were acquired using a confocal microscope Zeiss LSM 800 and processed using Fiji software. LAMP1 immunofluorescence images were processed simultaneously for each experiment using Fiji software, to generate binarized masks that were subjected to particle analysis. Particles with 10 *μ*m^2^ or lower area and a circularity index of 0.60 or more were considered as small lysosomes. Particles with values higher than 10 *μ*m^2^ of area and a circularity index of 1 or less were considered as enlarged lysosomes.

### 2.5. SA–*β*-Galactosidase Staining

The *β*-galactosidase activity was analyzed following the protocol described previously [26]. Cells fixed with 2% formaldehyde+0.2% glutaraldehyde and brain sections previously fixed with 4% paraformaldehyde were washed with PBS 1× and stained with the staining solution containing 20 mg/ml of X-gal (IB02260, IBI SCIENTIFIC, Peosta, IA, USA) in dimethylformamide, 0.2 M citric acid/sodium phosphate buffer pH = 6, 100 mM potassium ferrocyanide, 5 M sodium chloride, and 1 M magnesium chloride. Sections were incubated for 12 h and cells for 24 h at 37°C.

#### 2.5.1. Brain Sections

To corroborate staining, the complete regions of the hippocampus and cerebral cortex were imaged at 10× using an inverted Nikon Eclipse Ti-U microscope and accompanying NIS Elements, Basic Research (Nikon Instruments Inc. ®, NY, USA) software. In order to obtain better resolution images of each focal plane, CA3 and hilus of hippocampus and perirhinal cortex were imaged at 40× using transmitted light illumination of a confocal microscope Zeiss LSM 800. Images were processed and quantified using Fiji software. The percentage of SA–*β*-gal positive was quantified using images of each hippocampal region and perirhinal cortex from both hemispheres of three different animals per group. All images were analyzed using binarized masks, IsoData threshold, and particle analysis in the Fiji software.

#### 2.5.2. Primary Culture

Confocal detection of X-gal in cultured neurons was performed as previously described (Levitsky et al. 2013) and quantified using confocal images acquired with 63× objective. All images were processed simultaneously for the three independent experiments using Fiji–ImageJ, to generate binarized masks and define the IsoData threshold. Then, these masks were subjected to particle analysis of the ImageJ macro. Finally, the percentage of the SA–*β*-gal-positive area was divided by the total number of nuclei of each experiment.

### 2.6. Statistics

All data were analyzed and graphed with Prism 6 (GraphPad Software Inc. La Jolla, CA, USA). Specific tests were performed according to each experimental design.

Western blot analysis for in vivo samples was performed using one-way ANOVA and Dunnet as the posthoc test. Western blot analysis for in vitro samples was performed using two-way ANOVA followed by Holm-Sidak's posthoc test. In vivo pixel density analysis and SA-*β*-gal-positive area were performed using unpaired *t*-test student or one-way ANOVA analysis, followed by Sidak's multiple comparison tests, as indicated in each figure. Pearson's correlation coefficient was included. In vitro pixel density of cortical neurons was analyzed with one-way ANOVA analysis, followed by Tukey's or Sidak's multiple comparison test as indicated in each figure. Lysosome morphologies were compared using ordinary one-way ANOVA analysis, followed by Sidak's multiple comparison test.

## 3. Results

### 3.1. CRM1 Accumulates in Aged Neurons in the Hippocampus and Perirhinal Cortex

**Figure 1:**
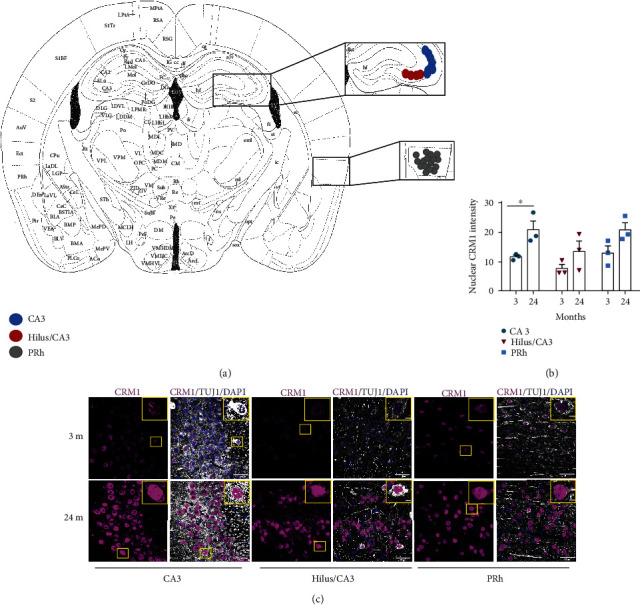
CRM1 accumulates in neurons during brain aging. (a) Anatomic diagram to locate the regions analyzed along the work. (b) Graph shows pixel density of nuclear CRM1 in neurons of mice at the indicated ages and brain regions. Bars represent the mean ± SEM from three independent experiments, with significant differences determined by unpaired *t*-test Student; ^∗^*p* < 0.05. Three or four fields per region were analyzed, and 70-100 neurons per region per animal were counted. (In total, we counted for CA3, *n* = 300 neurons of each age; for hilus, *n* = 274 neurons from 3 months old and *n* = 263 neurons from 24 months old; and for PRh, *n* = 115 neurons from 3 months old and *n* = 139 neurons from 24 months old). (c) Representative images of immunofluorescences to detect CRM1 in neurons (expressing TUJ1), in the hippocampus (CA3 and hilus) ,and perirhinal cortex (PRh) from young (3 months-old) and old (24 months-old) mice used for the quantification shown in (b). Scale bars represent 30 *μ*m. Nuclei were stained with DAPI. Squares indicate the magnified area shown in insets.

To ascertain whether CRM1 level increases during aging in the mouse brain, we compared CRM1 expression in the hippocampus and cortex of young and old mice. We chose those regions because they play an important role in learning and memory and undergo functional and structural alterations during physiological aging and age-related neurodegenerative diseases, like Alzheimer's disease [27, 28]. We observed an increase of CRM1 in neurons in the CA3 region and hilus/CA3 areas of the hippocampus, as well as in neurons in the perirhinal cortex (PRh), when brains from 24 months old mice were compared with those of 3 months old mice (Figure 1). To rule out that the increase of CRM1 observed was due just to an increase in the size of old neurons, we measured the area of both nucleus and cytoplasm of neurons from 3 and 24 months old brains. As show in Supplementary Figure 1, the average size of neurons does not change significantly with age. The age-associated increased level of CRM1 was confirmed by western blot analysis by comparing whole hippocampus and cortex lysates from 24-28, 14-17, and 3-6 months old mice (Supplementary Figure 2).

### 3.2. Neurons Expressing High Level of CRM1 in Old Brains Display Enhanced Nuclear Export of TFEB and STAT3 and Accumulate p62/SQSTM1

**Figure 2:**
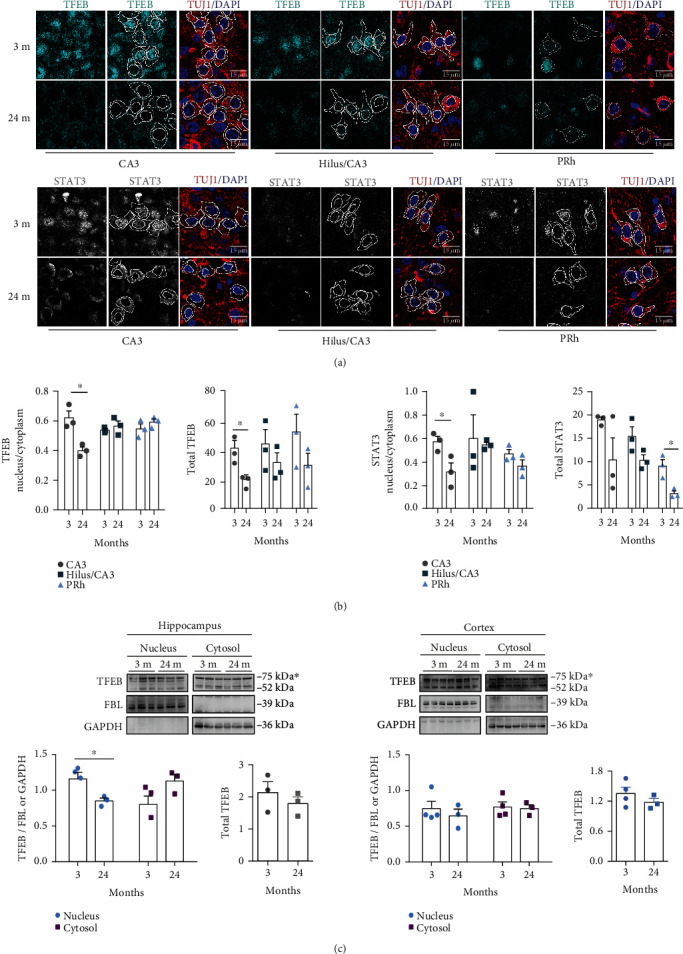
Enhanced TFEB and STAT3 nuclear export in CA3 neurons during brain aging. (a) Immunofluorescence analysis was carried out to detect TFEB and STAT3 in neurons (expressing TUJ1) of the indicated brain regions, collected from 3 and 24 months old mice. Scale bars, 15 *μ*m. Nuclei were stained with DAPI. (b) Pixel density of TFEB or STAT3 signals in nuclear and cytoplasmic areas was quantified; white lines in panel (a) exemplify delineated areas. Left graphs represent the nuclear/cytoplasmic ratio found in each region and age; right graphs represent the total signal (nuclear plus cytoplasmic signal); bars represent the mean ± SEM. Three or four fields per region per animal were analyzed. The collective number of neurons counted was CA3, *n* = 150 neurons from 3 months old and *n* = 120 neurons from 24 months old; for hilus, *n* = 86 neurons from 3 months old and *n* = 70 neurons from 24 months old; and for PRh, *n* = 75 neurons from 3 months old and *n* = 77 neurons from 24 months old. Significant differences were determined by unpaired *t*-test Student ^∗^*p* < 0.05. (c) Subcellular distribution of TFEB was analyzed by western blot of cytoplasmic and nuclear fractions from the hippocampus and cortex of mice at the indicated ages. GAPDH and fibrillarin (FBL) detection was used as cytoplasmic and nuclear markers, respectively. Left graphs represent the mean of densitometry analysis of the 75 KDa band of TFEB, and right graphs represent total TFEB (nuclear plus cytoplasmic densitometry) from at least three animals per age group, expressed in arbitrary units. Bars correspond to the mean ± SEM from three independent experiments, with significant differences determined by unpaired *t*-test Student; ^∗^*p* < 0.05.

To determine whether elevated CRM1 level in old brains is reflected in increased CRM1 activity, we analyzed the subcellular distribution of TFEB and STAT3, proteins whose nuclear export depend on CRM1 [24, 25, 29]. A decrease in the nuclear labeling of both TFEB and STAT3 was observed in CA3 and PRh neurons from 24 months old mice, compared with 3 months mice (Figure 2). We quantified the partition of nuclear vs. cytoplasmic signal and found in the CA3 region an increased export of TFEB statistically significant and a trend of increased export of STAT3 in the PRh region (Figure 2(b)).

Consistently, a reduction of TFEB in the nuclear fraction of the hippocampus, with its concomitant increase in the cytoplasmic fraction, was found by western blot analysis in 24 months old mice. Such enrichment in the cytoplasm was not observed in the cytoplasmic fraction from the cortex (Figure 2(c)). The overall reduction of the TFEB expression noticed previously during the analysis per cel by immunofluorescence was not detectable by western blot, when the whole hippocampus is lysed (Figure 2(c)).

Collectively, our data imply that increased the CRM1 level corresponds to its enhanced activity in CA3.

**Figure 3:**
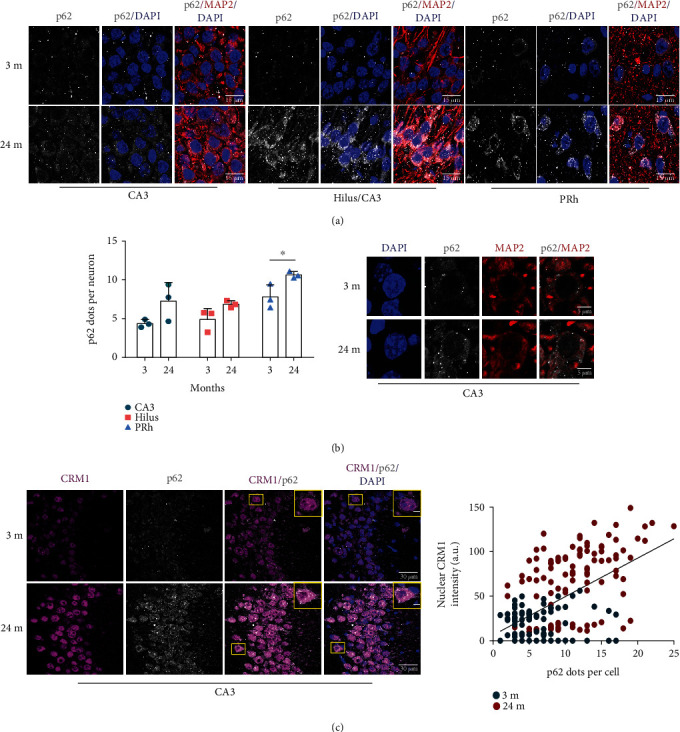
Accumulation of p62/SQSTM1 correlated with high nuclear level of CRM1 in neurons of 24 months old mice. (a) Immunofluorescence analysis to detect p62/SQSTM1 in neurons (expressing MAP2) at the indicated brain regions from 3 or 24 months old mice. Scale bars, 15 *μ*m. Nuclei were stained with DAPI. (b) Graph shows quantification of p62/SQSTM1 dots per neuron. Bars correspond to the mean ± SEM from three independent experiments, with significant differences determined by unpaired *t*-test Student; ^∗^*p* < 0.05. Three or four fields per region per animal were analyzed. The total number of neurons counted was CA3, *n* = 187 from 3 months old and *n* = 201 from 24 months old; hilus, *n* = 161 from 3 months old and *n* = 285 from 24 months old; and PRh, *n* = 113 from 3 months old and *n* = 124 from 24 months old. Right, an amplified image representative of the neurons was counted. (c) Coimmunodetection of CRM1 and p62/SQSTM1 in the CA3 region of the hippocampus from 3 or 24 months old mice. Scale bars, 30 *μ*m. Nuclei were stained with DAPI. The plot shows a linear regression analysis that positively correlates the nuclear CRM1 intensity with p62/SQSTM1 dots in cells (*r* = 0.5985, *n* = 100 neurons from 3 months; *n* = 102 neurons from 24 months, *p* < 0.0001). Squares indicate the magnified area shown in insets.

**Figure 4:**
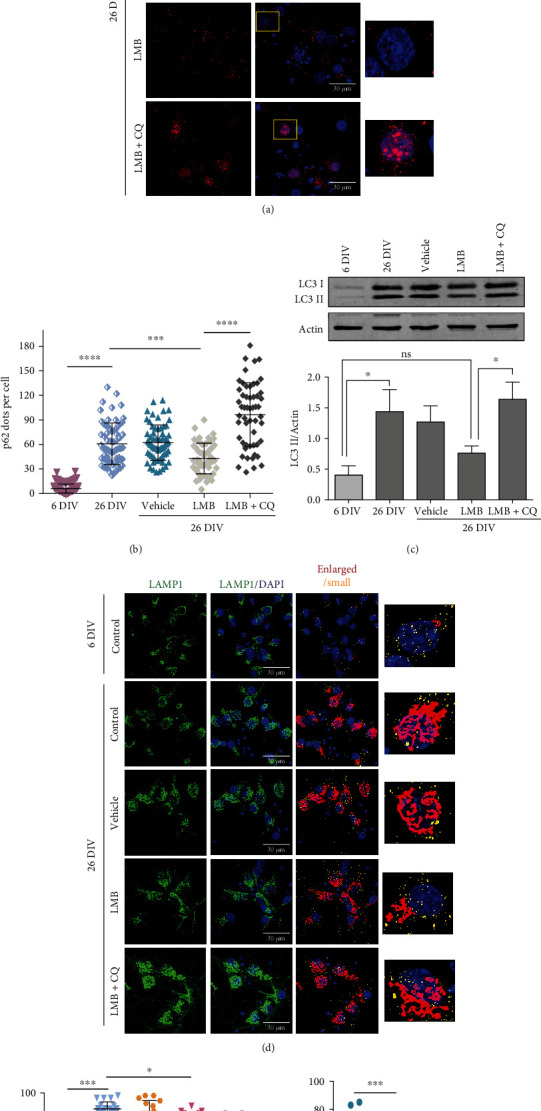
CRM1 inhibition restored the autophagic flux in cultured 26 DIV neurons. Cortical neurons cultured during 6 and 26 DIV were treated for 4 days (starting at 22 DIV) with 5 nM LMB, or with the vehicle alone, or with 5 nM LMB and 20 *μ*M chloroquine (CQ) for 4 h. (a) Immunofluorescence analysis to detect p62/SQSTM1. Scale bars, 30 *μ*m. Squares indicate the magnified area shown in insets. (b) Graph shows quantification of p62/SQSTM1 dots per cell. Bars correspond to the mean ± SD from three independent experiments, with significant differences calculated by ordinary one-way ANOVA analysis, with Tukey's multiple comparison test; ^∗∗∗^*p* < 0.001; ^∗∗∗∗^*p* < 0.0001. (c) Western blot detects LC3 and ACTIN. Graph shows the densitometric quantification of LC3-II normalized against ACTIN. Bars represent the mean ± SEM from three independent experiments, with significant differences determined by two-way ANOVA followed by Holm-Sidak's multiple comparison test; ^∗^*p* < 0.05; ns: no significant. (d) Immunofluorescence analysis to detect LAMP1. (e) The number of enlarged lysosomes per cell is shown. (f) The number of small lysosomes per cell is plotted. The quantification method is described in the methods section. Bars represent the mean ± SD, with significant differences calculated by ordinary one-way ANOVA analysis, with Sidak's multiple comparison test; ^∗^*p* < 0.05; ^∗∗∗∗^*p* < 0.0001.

Since we had previously observed that the autophagic flux is impaired in senescent neurons in vivo and in vitro [13], we next wondered whether decreased nuclear localization of TFEB and STAT3 in old neurons might impair the autophagic flux [30]. Consistent with this idea, we observed accumulation of the autophagy adaptor protein p62/SQSTM1 in 24 months old brain regions (Figure 3(a)), being statistically significant in PRh cortex neurons (Figure 3(b)). A positive correlation between p62/SQSTM1 accumulation and increased CRM1 expression was corroborated by double-labeling immunofluorescence assays in CA3 neurons of 24 months old brains (Figure 3(c)). Although no statistically significant change was observed for GFP-LC3 intensity (Supplementary Figure 3), we confirmed that autophagy dysfunction occurs in senescent neurons in vitro, as we observed that they accumulate p62/SQSTM1, LC3, and enlarged lysosomes (Figure 4). Taken together, these findings imply that cytoplasmic localization of TFEB and STAT3 in aged neurons, due likely to enhanced CRM1 activity, could lead to autophagic flux impairment.

### 3.3. Neurons with Senescent Features Increase in Old Mice and Correlate with Increased CRM1 Expression

**Figure 5:**
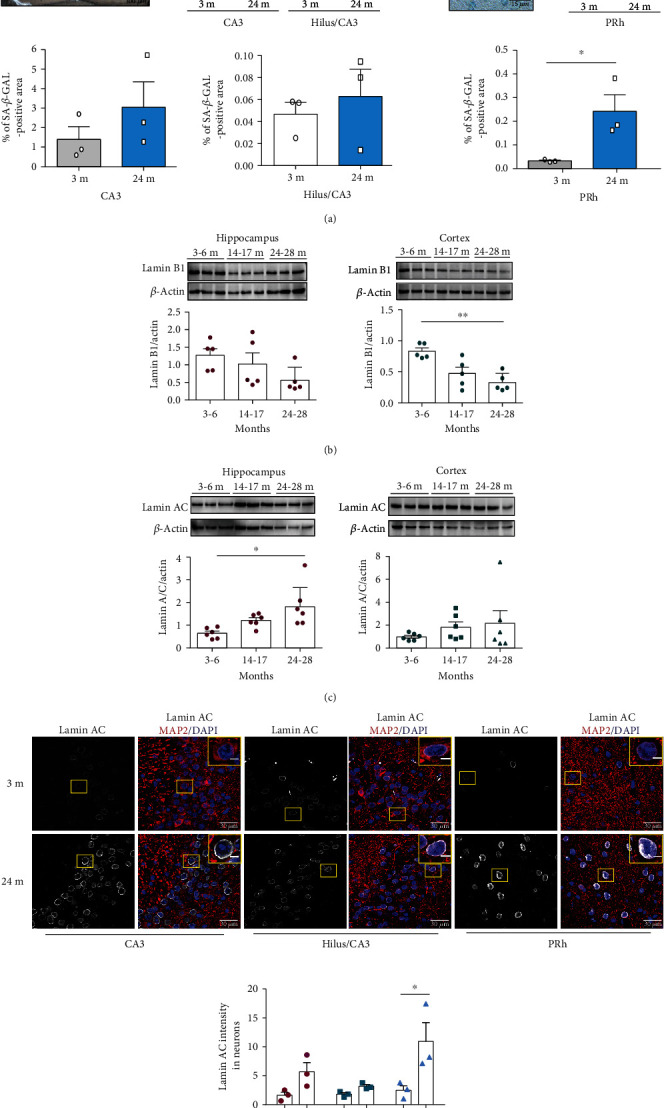
Neurons with senescence features accumulate in the hippocampal and cortical regions in 24 months old mice. (a) SA-*β*-gal activity was quantified in the hippocampal and cortical regions of 3 and 24 months old mice as indicated in the methods section. Graphs show the percentage of the SA-*β*-gal-positive area in each brain region at the indicated mice ages. Bars correspond to the mean ± SEM from three independent experiments, with significant differences determined by unpaired *t*-test Student; ^∗^*p* < 0.05. (b) Western blot analysis showing the expression of lamin B1 and (c) lamin A/C in the hippocampus and cortex of the indicated mice ages. Graphs show the densitometric analysis of immunoblot autoradiograms to determine lamin B1 and lamin A/C protein expression from five or six animals per age group (each dot represents an animal). Data represent the mean ± SEM, with significant differences calculated by one-way ANOVA and Dunnet's posthoc test; ^∗^*p* < 0.05; ^∗∗^*p* < 0.01. (d) Immunofluorescence analysis to detect lamin A/C in neurons (expressing MAP2) of the indicated brain regions from 3 and 24 months old mice. Scale bars, 30 *μ*m. Nuclei were stained with DAPI. Squares indicate the magnified area shown in insets. Graph shows pixel density of lamin A/C in the indicated brain regions and mice ages. Bars represent the mean ± SEM from three independent experiments, with significant differences calculated by two-way ANOVA analysis, with Sidak's multiple comparison test; ^∗^*p* < 0.05.

Since CRM1 overexpression promotes cellular senescence in normal fibroblasts [21], we wondered whether enhanced CRM1 activity might also contribute to neuronal senescence during physiological aging. To this end, we examined senescent features in neurons of old mice brains. We detected an increased number of SA-*β*-gal-positive neurons in the hippocampus and PRh cortex of 24 months old mice brains (Figure 5(a)). Senescent cells have aberrant nuclear morphology and altered distribution of nuclear envelope proteins [21]; loss of lamin B1 and its receptor LBR1 induces senescence [17], while lamin A/C stabilization leads to polycomb repressive complex 1 (PRC1) degradation, which allows *Cdkn2a* expression [16]. In line with this, we found a significant decrease of lamin B1 in both the hippocampus and PRh cortex of 14-17 and 24-28 months old mice brains (Figure 5(b)), as well as an increase of lamin A/C in the hippocampus neurons of 24-28 months old mice brains (Figure 5(c)). In concordance, increased lamin A/C immunolabeling was observed in the CA3 and PRh neurons of aged mice (Figure 5(d)).

**Figure 6:**
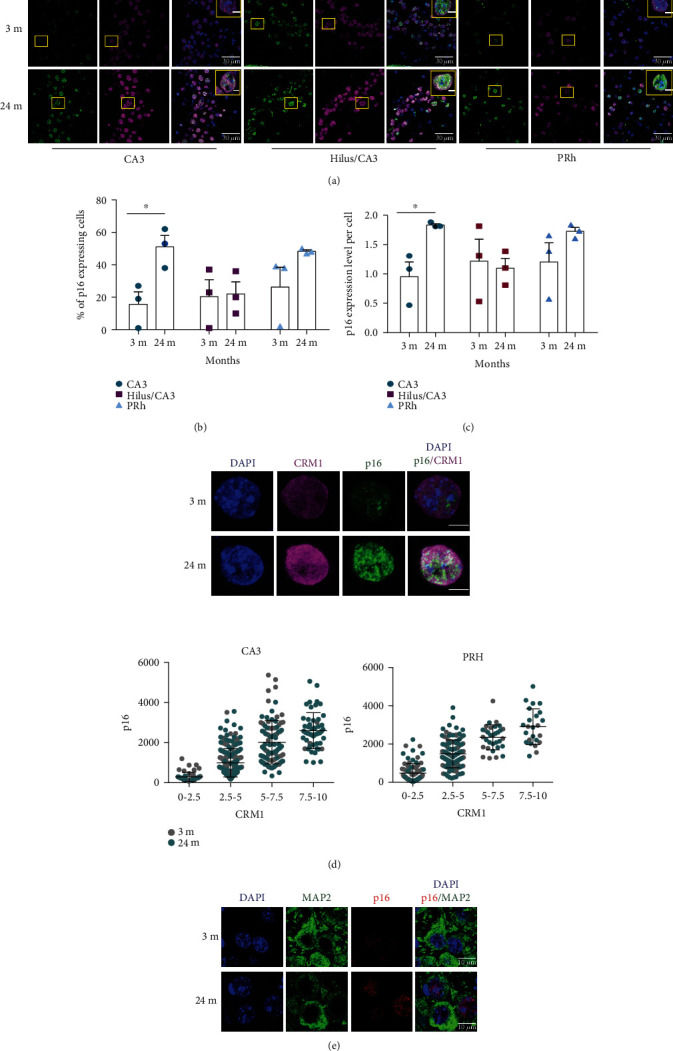
Brain cells with increased expression of p16^INK4A^ also express elevated levels of CRM1 in the CA3 and PRh cortex of 24 months old mice. (a) Immunofluorescence analysis to detect p16^INK4A^ and CRM1 in the hippocampus (CA3 and hilus) and PRh cortex of young (3 months old) and old (24 months old) mice brains. Nuclei were stained with DAPI. Squares indicate the magnified area shown in insets. Scale bars represent 30 *μ*m. (b) Graph shows the percentage of cells expressing detectable levels of p16^INK4A^. Data correspond to the mean ± SEM, with significant differences calculated by *t*-test Student; ^∗^*p* < 0.05. (c) Graph shows the pixel density of nuclear p16^INK4A^. Results show the mean ± SEM from three separate experiments, with significant differences calculated by *t*-test Student; ^∗^*p* < 0.05. For (b) and (c), the number of cell counted from 3 brains was CA3, *n* = 233 from 3 months old and *n* = 221 from 24 months old; hilus, *n* = 134 from 3 months old and *n* = 159 from 24 months old; and PRh, *n* = 168 from 3 months old and *n* = 151 from 24 months old. (d) Representative magnified images to visualize immunostaining of p16^INK4A^ and CRM1 are shown. Scale bars represent 5 *μ*m. Bottom, quantification of the nuclear p16^INK4A^ pixel density was plotted against the nuclear CRM1 pixel density in the CA3 and PRh cortex cells of the indicated mice ages. Bars represent the mean ± SEM (from 3 brains, the number of cell counted was CA3, *n* = 190 from 3 months old and *n* = 200 from 24 months old and PRh, *n* = 125 from 3 months old and *n* = 132 from 24 months old). (e) Immunofluorescence analysis to detect p16^INK4A^ in neurons (expressing MAP2) in the CA3 region of 3 and 24 months old brains. Scale bars represent 10 *μ*m.

We then analyzed whether the same cells that express p16*^INK4A^*, a mediator of cellular senescence [31], also have high CRM1 expression. Interestingly, increased immunostaining of p16*^INK4A^* and CRM1 concurred in the CA3 and PRh neurons of aged mice (Figures 6(a) and 6(b)). Even though there were some cells expressing p16*^INK4A^* at 3 months old, they had lower expression level than cells expressing p16*^INK4A^* in 24 months old brains, particularly in the CA3 region (Figure 6(c)). We found indeed a direct correlation between the immunolabeling intensity of p16*^INK4A^* and CRM1 in the CA3 and PRh brain cells of 24 months old mice (Figure 6(d)). We verified that neurons in particular express high levels of p16*^INK4A^* in the CA3 region at 24 months old brains (Figure 6(e)). These findings led us to speculate that enhanced CRM1 activity might trigger neuronal senescence.

### 3.4. Pharmacological Inhibition of CRM1 Improves Autophagic Flux and Reduces SA-*β*-GAL Activity in Senescent Neurons In Vitro

**Figure 7:**
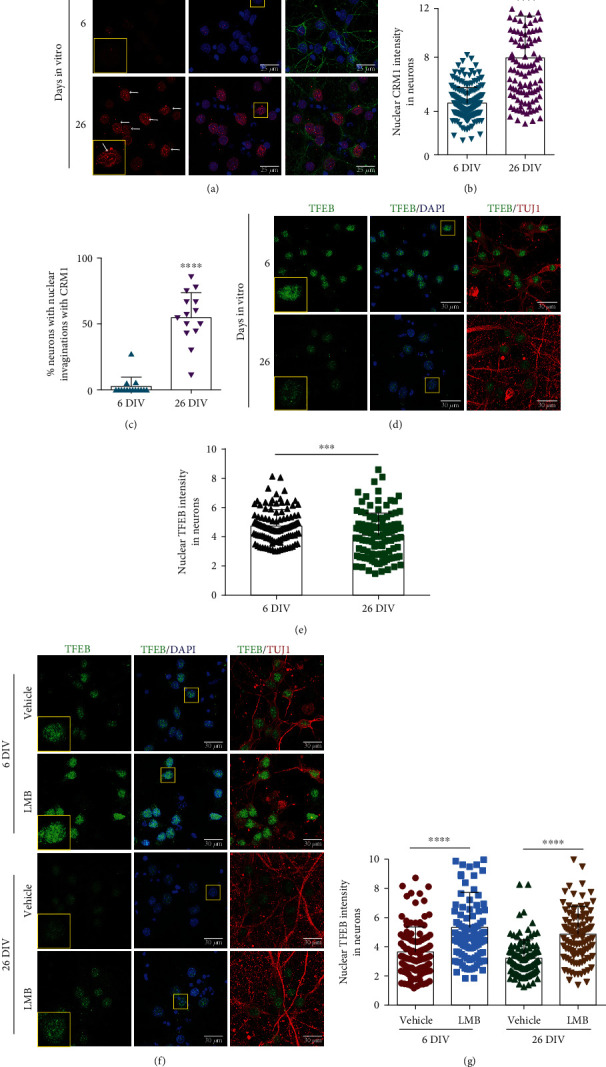
CRM1 accumulates in cultured senescent neurons and its pharmacological inhibition restored TFEB nuclear localization. (a) Immunofluorescence analysis was carried out to detect CRM1 in primary cortical neurons (expressing TUJ1) cultured during the indicated DIV. Arrows indicate CRM1 enrichment in nuclear envelope invaginations. Scale bars, 25 *μ*m. (b) Graph shows pixel density of nuclear CRM1 in cortical neurons incubated during the indicated DIV. (c) Graph shows the percentage of neurons with CRM1-containing nuclear invaginations over total cells. Bars represent the mean ± SD (*n* = 100 cells per experimental group, from three independent experiments) with significant differences determined by unpaired *t*-test Student; ^∗∗∗∗^*p* < 0.0001. (d) Immunofluorescence analysis to detect TFEB in neurons (expressing TUJ1) in primary cortical cells cultured during the indicated DIV. Scale bars, 30 *μ*m. (e) Graph shows the pixel density of nuclear TFEB in cortical neurons incubated during the indicated DIV. Bars represent the mean ± SD (*n* = 100 cells per experimental group, from three independent experiments), with significant differences determined by unpaired *t*-test Student; ^∗∗∗^*p* < 0.001. (f) Primary cortical cells cultured during 6 or 26 DIV were treated for 24 h with 5 nM LMB or the vehicle alone and immunolabeled to detect TFEB. (g) Graph represents the pixel density of nuclear TFEB. Bars correspond to the mean ± SD (*n* = 100 cells per experimental group, from three independent experiments), with significant differences determined by unpaired *t*-test Student; ^∗∗∗∗^*p* < 0.0001. Squares indicate the magnified area shown in insets (a, d, f).

To analyze whether enhanced CRM1 activity contributes to neuronal senescence through perturbation of the autophagic flux, we used a long-term culture of rat primary cortical neurons. Neurons at 26 days in vitro (DIV) display autophagy dysfunction and cellular senescence characteristics, recapitulating then features of physiological aging [13, 19]. We observed that neurons at 26 DIV accumulated CRM1, and that around half of them displayed nuclear envelope invaginations, a senescent feature, compared with 6 DIV neurons (Figures 7(a)–7(c)). Consistent with enhanced CRM1 activity, reduced nuclear immunolabeling of TFEB (a CRM1 target) was specifically observed in 26 DIV senescent neurons (Figures 7(d) and 7(e)). As TFEB is a main positive modulator of autophagy, we hypothesized that treatment of neuron cultures with the CRM1-specific inhibitor leptomycin B (LMB) may accumulate TFEB in the nucleus and consequently restore proper autophagic flux in 26 DIV neurons. Supporting this notion, a significant nuclear enrichment of TFEB was found in the 6 DIV and 26 DIV neuronal cultures upon treatment with LMB, compared with cultures treated with the vehicle alone in 26 DIV neurons (Figures 7(f) and 7(g)). Remarkably, pharmacological inhibition of CRM1 prevented the accumulation of p62/SQSTM1 and LC3-II levels in 26 DIV neurons, compared with control 6 DIV neurons and vehicle-treated 26 DIV neurons (Figures 4(a)–4(c)). The LMB-mediated decrease of p62/SQSTM1 and LC3-II was blocked in the presence of chloroquine, an inhibitor of autophagolysosome maturation. We next analyzed lysosome morphology, a critical parameter of autophagy function. As shown in Figures 4(d)–4(f), senescent neurons exhibited enlarged LAMP1-stained lysosomes, which are characteristic of low degradation rate and accumulation of protein aggregates [32–34], while LMB-treated senescent neurons showed significant decrease of enlarged lysosomes, with concomitant increase of functional, small-size lysosomes, compared with vehicle-treated senescent neurons. Collectively, our results imply that pharmacological CRM1 inhibition restored the autophagy flux through improvement of TFEB activity.

**Figure 8:**
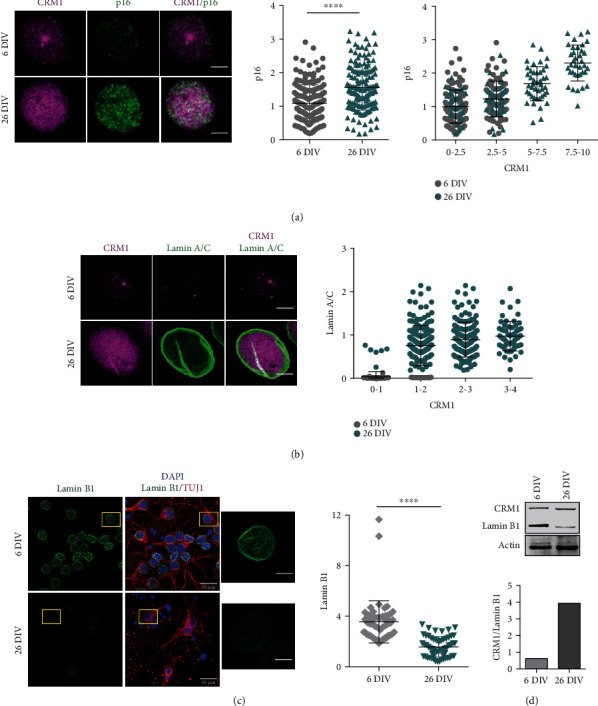
Elevated level of CRM1 correlated with neuronal senescence markers in vitro. (a) Immunofluorescence analysis to detect p16^INK4A^ and CRM1 in primary cortical cells cultured during 6 and 26 DIV. Scale bars represent 30 *μ*m. Nuclei were stained with DAPI. Left graph shows the pixel density of nuclear p16^INK4A^ in cortical cells; right graph shows a correlation between p16^INK4A^ and CRM1 expression levels. Bars represent the mean ± SD from three independent experiments, with significant differences determined by unpaired *t*-test Student; ^∗∗∗∗^*p* < 0.0001. Notice that cells at 26 DIV have higher expression of CRM1 and p16^INK4A^ compared with 6 DIV. (b) Immunofluorescent double staining for CRM1 and lamin A/C. Scale bars represent 5 *μ*m. Quantification of lamin A/C intensity versus the CRM1 intensity is plotted. Notice that cells at 26 DIV have higher expression of CRM1 and lamin A/C. Bars represent the mean ± SD from 3 independent experiments. (c) Immunofluorescence to detect lamin B1 in primary cortical neurons (expressing TUJ1) of 6 and 26 DIV. Scale bars, 30 *μ*m. Squares indicate the magnified area shown at the right; scale bars 5 *μ*m. Graph represents the signal fluorescence intensity of nuclear lamin B1. Bars represent the mean ± SD from 3 independent experiments with significant differences determined by unpaired *t*-test Student; ^∗∗∗∗^*p* < 0.0001. Each dot in each graph represents a cell measured from 3 independent experiments. (d) Western blot of total protein extracts from cortical cells cultured for 6 or 26 DIV. The ratio of CRM1 over the lamin B1 expression (both previously normalized by actin expression) is compared in the graph below, *n* = 2.

**Figure 9:**
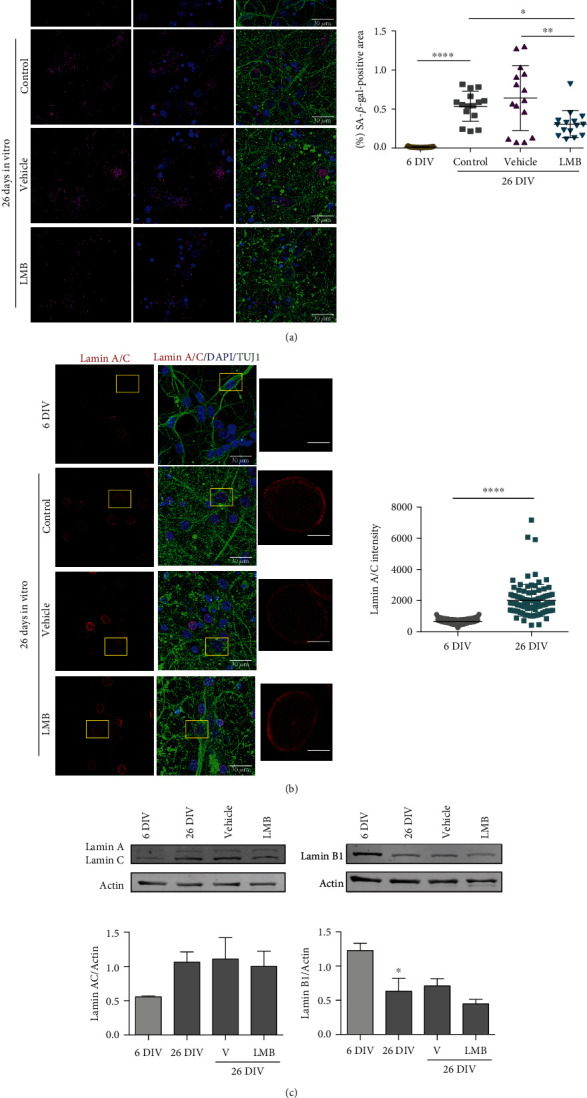
CRM1 inhibition reduced SA-*β*-gal activity but did not restored nuclear lamin alterations in senescent neurons in vitro. (a) Detection of SA-*β*-gal activity (by confocal microscopy) in neurons (expressing TUJ1) in primary culture of cortical cells of 26 DIV, treated for 4 days (starting at 22 DIV) with 5 nM LMB or the vehicle alone. Scale bars, 30 *μ*m. Graph shows the percentage of the SA-*β*-gal-positive area. Five fields from three independent experiments were quantified. Bars represent the mean ± SD from 3 independent experiments with significant differences determined by ordinary one-way ANOVA analysis, with Holm-Sidak's multiple comparison test; ^∗^*p* < 0.05; ∗∗*p* < 0.01; ^∗∗∗∗^*p* < 0.0001. Notice that LMB treatment significantly reduced SA-*β*-gal-positive area. (b) Immunofluorescence to detect lamin A/C in neurons (expressing TUJ1) in primary culture of cortical cells of 26 DIV, treated for 4 days (starting at 22 DIV) with 5 nM LMB or the vehicle alone. Scale bars, 30 *μ*m. Squares indicate the magnified area shown in insets, scale bars 5 *μ*m. Graph shows fluorescence intensity of nuclear lamin A/C in neurons cultured 6 or 26 DIV. Bars represent the mean ± SD from three independent experiments, with significant differences determined by unpaired *t*-test Student; ^∗∗∗∗^*p* < 0.0001. (c) Western blot analysis showing the expression of lamin A/C or lamin B1 in primary culture of cortical cells of 6 or 26 DIV treated or not with LMB or the vehicle alone. Bars represent the mean ± SEM from three independent experiments, with significant differences determined by ordinary one-way ANOVA and Holm-Sidak's multiple comparison test; ^∗^*p* < 0.05.

Next, we explored the notion that enhanced CRM1 activity might be mechanistically linked to neuronal senescence. To approach this, we analyzed the response of 6 DIV and 26 DIV neuron cultures to LMB treatment and evaluated four markers of cellular senescence, namely, p16*^INK4A^* induction, lamin A/C stabilization, lamin B1 depletion, and SA-*β*-Gal activity. As observed above in vivo, the immunostaining of p16*^ink4a^* and lamin A/C increased in correlation with that of CRM1 in 26 DIV cortical neurons (Figures 8(a) and 8(b)). Furthermore, we observed also a decrease of lamin B1 immunolabeling (Figure 8(c)) and high of SA-*β*-gal activity (Figure 9(a)), confirming the senescent phenotype of the 26 DIV neuronal culture. A CRM1 increase concomitant with lamin B1 depletion in 26 DIV neuronal culture was confirmed by western blot (Figure 8(d)).

Interestingly, attenuation of CRM1 activity using LMB treatment for the last four days of culture (from 23 DIV to 26 DIV) reduced SA-*β*-gal activity (Figure 9(a)) in 26 DIV neurons; however, no changes in lamin A/C and lamin B1 levels were observed by immunofluorescence (Figure 9(b)) nor by western blot (Figure 9(d)).

## 4. Discussion

This work was aimed at determining whether aging-associated enhanced activity of CRM1 is the mechanism that functionally connects autophagy dysfunction with neuronal senescence. Trafficking of proteins between the nucleus and cytoplasm is modulated by the nuclear pore complex (NPC) and karyopherins that transport proteins in and out of the nucleus. Importins drive the nuclear import of proteins with nuclear localization sequences, while exportins mediate the nuclear export of NES-containing proteins, being CRM1 the major mammalian export protein. As TFEB is a master modulator of autophagy and lysosomal biogenesis [23, 24], whose nuclear export is mediated by CRM1 via recognition of the NES localized in the N-terminal domain of TFEB [35], we reasoned that enhanced CRM1 activity might cause depletion of nuclear TFEB, which in turn can perturb autophagy and ultimately drive neurons to senescence. Consistently, we found that CRM1 protein levels increased in the hippocampus and cortex of aged mice, concomitantly with a decrease of nuclear TFEB, implying that increased CRM1 activity improved nuclear export of TFEB. The CRM1-mediated nuclear depletion of TFEB resulted in impaired autophagic flux in the CA3 region of 24 months old mice brains, as shown by accumulation of p62/SQSTM1 in neurons that overexpressed CRM1. Strengthening our hypothesis, CRM1-driven decrease of nuclear TFEB occurred in conjunction with the accumulation of p62/SQSTM1 and the presence of LAMP1-stained enlarged lysosomes in 26 DIV cultured neurons as well.

Alterations in nuclear shape and nuclear membrane function are associated with several deleterious changes such as disrupted nucleocytoplasmic function and commonly occur in physiological aging [36]. Tau-mediated dementias are also accompanied by dysfunction in neuronal nucleocytoplasmic transport [37], and alterations of the nuclear lamina have been observed in postmortem Alzheimer's disease brain [38] and several models of Huntington's disease [39]. We observed that CRM1 is enriched in nuclear envelope invaginations of senescent neurons, which might potentially compartmentalize NPC and increase then the kinetics of CRM1-driven nuclear export, as has been observed for mRNA export in tau-induced nuclear envelope invaginations [40]. Furthermore, dysfunctional autophagy contributes to the formation of neuronal aggregates and characteristic of neurodegeneration, which are known to damage NPC [41], with the obvious effect on protein trafficking across the NPC. Nonetheless, enhanced activity of CRM1 in skin fibroblasts of HGPS patients is carried out by transcriptional upregulation of CRM1 [21]. Further experiments to analyze CRM1 gene regulation during brain aging are required to approach this question.

Pharmacological interventions that attenuate CRM1 activity reduce neuronal death and restore nucleocytoplasmic transport in either a model of Huntington's disease [39], a model of amyotrophic lateral sclerosis and frontotemporal dementia [42], and a *Drosophila* model of tauopathies [40]. Thus, in this work, we counteracted the increased activity of CRM1 by treating 26 DIV cultured neurons with LMB1 (a specific CRM1 inhibitor), as an attempt to accumulate TFEB in the nucleus and consequently activate its transcriptional function. Strikingly, pharmacological inhibition of CRM1 restored proper autophagic flux in 26 DIV neurons, as shown by reduced p62/SQSTM1 and LC3 accumulation in LMB-treated cells, and the presence of small lysosomes. Improving autophagy by modulating CRM1 activity appears to delay senescence, because a decrease in SA-*β*-gal activity was observed in LMB-treated 26 DIV neurons. Repartitioning of other CRM-target proteins in the nucleus, such as STAT3, may also contribute to improve autophagy and delay senescence. We found no restoration of lamin A/C nor lamin B levels in response to LMB, suggesting that the senescent phenotype was not fully reverted in neurons once it was established. This finding seems to be in contrast with the previous observation that LMB treatment restores the lamin B1 expression in HGPS senescent fibroblasts [21]. We argue that a reduction in the number of senescent fibroblasts upon LMB treatment could be explained by their proliferative nature, which is in contrast with neurons. Since a subpopulation of fibroblasts are not yet senescent at the moment LMB is added, the reduction in the number of senescent fibroblasts could be due to a prevention of new geroconversion of proliferating cells, instead of a reversion of the senescent phenotype. Further experiments are needed to distinguish between prevention and reversion of cellular senescence.

DNA damage induces a regulated enhanced nucleocytplasmic export to promote genome stability; our findings suggest that it might become dysfunctional with aging. When DNA is damaged, some DNA repair proteins such as BRCA1, MRE11, RAD50, and NBS, as well as damaged DNA itself, are translocated from the nucleus to the cytoplasm by a CRM1-mediated nuclear export [43–46]. Cytoplasmic DNA is degraded by autophagy; otherwise, it induces cellular senescence [46]. Since DNA damage occurs continually, our findings of an age-associated exacerbated CRM1 activity and autophagy dysfunction could cause persistent cytoplasmic DNA promoting cellular senescence.

Taken together, our study demonstrates that age-associated enhanced activity of the CRM1-mediated nuclear export mechanism perturbs neuronal homeostasis by contributing to autophagy impairment, which in turn triggers neuronal senescence. Therefore, CRM1 may serve as a therapeutic target to prevent/ameliorate all these effects found in physiological brain aging and neurodegeneration.

## Data Availability

All data is included in the manuscript, either in the main text and figures, as well as in the Supplementary Information files that are submit alongside our manuscript.

## Supplementary Materials

Supplementary Materials Supplementary Figure 1: the cellular area of neurons in young and old mice is similar. The nuclear (a) and cytoplasmic (b) areas (expressed in *μ*m^2^) of neurons (identified by MAP2 expression) were quantified in the hippocampal and cortical regions of brains from 3 and 24 months old mice. The graph below each representative image expresses the mean ± SEM. No significant differences were found, analyzed by unpaired t-test Student. From 3 brains, the total number of neurons counted was CA3, *n* = 248 from 3 months old and *n* = 203 from 24 months old; hilus, *n* = 148 from 3 months old and *n* = 132 from 24 months old; and PRh, *n* = 133 from 3 months old and *n* = 133 from 24 months old. Representative neurons of the CA3 regions are shown. Scale bars represent 15 *μ*m. Supplementary Figure S2: CRM1 accumulates in the hippocampus and cortex with age. Western blot analysis showing the expression of the CRM1 protein in hippocampus and cortex of wild-type mice at the indicated ages. Graph represents the mean of densitometry analysis of western blot analysis from six animals per age group expressed in arbitrary units. Bars correspond to the mean ± SEM from six independent experiments, with significant differences determined by one-way ANOVA and Dunnet as posthoc test; ^∗∗∗^*p* < 0.001. Supplementary Figure S3: pattern distribution and expression level of GFP-LC3 did not change during brain aging. (a) Confocal images showing GFP-LC3 signal in neurons (expressing MAP2) of the hippocampus and perirhinal cortex (PRh) from 3 and 24 months old GFP-LC3 transgenic mice. (b) Graph represents pixel density of GFP-LC3 signal in the different brain regions analyzed in three brains per age. We noticed an increment of GFP-LC3 along axons in the PRh region, but quantifying GFP-LC3 intensity, we found no significant difference. Bars represent the mean ± SEM.
