# Supplementary material for: Enhanced Activity of Exportin-1/CRM1 in Neurons Contributes to Autophagy Dysfunction and Senescent Features in Old Mouse Brain
Source: Oxid Med Cell Longev. 2021 Aug 13;2021:6682336. doi: 10.1155/2021/6682336 (PMC8382534; doi:10.1155/2021/6682336)
Supplement: Supplementary Materials — Supplementary Figure 1: the cellular area of neurons in young and old mice is similar. The nuclear (a) and cytoplasmic (b) areas (expressed in μm2) of neurons (identified by MAP2 expression) were quantified in the hippocampal and cortical regions of brains from 3 and 24 months old mice. The graph below each representative image expresses the mean ± SEM. No significant differences were found, analyzed by unpaired t-test Student. From 3 brains, the total number of neurons counted was CA3, n = 248 from 3 months old and n = 203 from 24 months old; hilus, n = 148 from 3 months old and n = 132 from 24 months old; and PRh, n = 133 from 3 months old and n = 133 from 24 months old. Representative neurons of the CA3 regions are shown. Scale bars represent 15 μm. Supplementary Figure S2: CRM1 accumulates in the hippocampus and cortex with age. Western blot analysis showing the expression of the CRM1 protein in hippocampus and cortex of wild-type mice at the indicated ages. Graph represents the mean of densitometry analysis of western blot analysis from six animals per age group expressed in arbitrary units. Bars correspond to the mean ± SEM from six independent experiments, with significant differences determined by one-way ANOVA and Dunnet as posthoc test; ∗∗∗p < 0.001. Supplementary Figure S3: pattern distribution and expression level of GFP-LC3 did not change during brain aging. (a) Confocal images showing GFP-LC3 signal in neurons (expressing MAP2) of the hippocampus and perirhinal cortex (PRh) from 3 and 24 months old GFP-LC3 transgenic mice. (b) Graph represents pixel density of GFP-LC3 signal in the different brain regions analyzed in three brains per age. We noticed an increment of GFP-LC3 along axons in the PRh region, but quantifying GFP-LC3 intensity, we found no significant difference. Bars represent the mean ± SEM. [file 6682336.f1.pdf]

SUPPLEMENTARY MATERIAL

Enhanced activity of Exportin-1/CRM1 in neurons contributes to autophagy dysfunction and senescent features in old mouse brain.

Elisa Gorostieta-Salas[1], Daniel Moreno-Blas[1], Cristian Gerónimo-Olvera[2], Bulmaro Cisneros[3], Felipe A Court[2,4,5], Susana Castro-Obregón[1*].

Figure S1

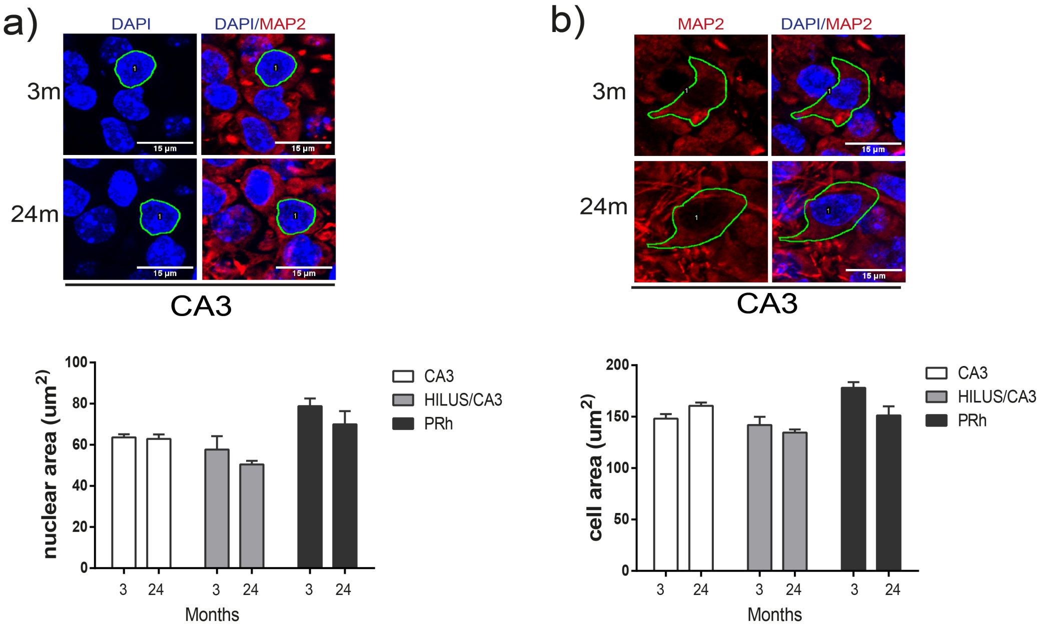

Supplementary Figure 1. The cellular area of neurons in young and old mice is similar. The nuclear **a)** and cytoplasmic **b)** areas (expressed in μm^2) of neurons (identified by MAP2 expression) were quantified in the hippocampal and cortical regions of brains from 3 and 24 months old mice. The graph below each representative image expresses the mean ± SEM. No significant differences were found, analyzed by unpaired t-Test Student. From 3 brains, the total number of neurons counted was: CA3, n=248 from 3 months-old and n=203 from 24 months-old; Hilus, n=148 from 3 months-old and n=132 from 24 months-old; PRh, n=133 from 3 months-old and n=133 from 24 months-old. Representative neurons of the CA3 regions are shown. Scale bars represent 15 μm.

Figure S2

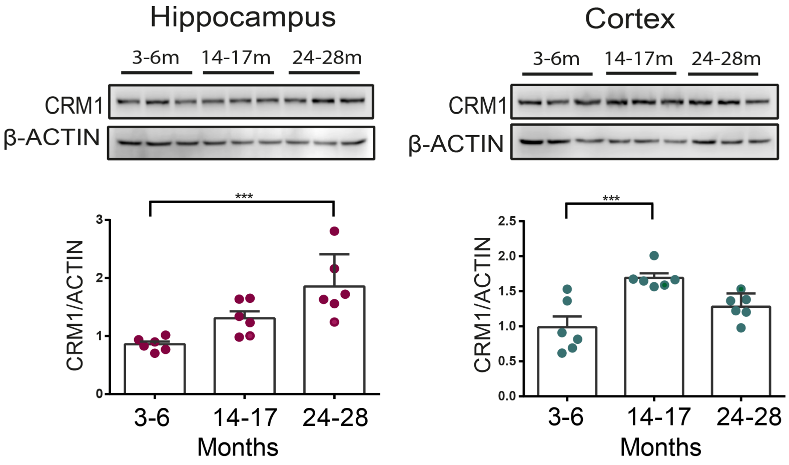

Supplementary Figure S2. CRM1 accumulates in hippocampus and cortex with age. Western blot analysis showing the expression of CRM1 protein in hippocampus and cortex of wild type mice at the indicated ages. Graph represents the mean of densitometry analysis of Western blot analysis from six animals per age group expressed in arbitrary units. Bars correspond to the mean ± SEM from six independent experiments, with significant differences determined by One-way Anova and Dunnet as *post hoc* test; ***p <0.001.

Figure S3

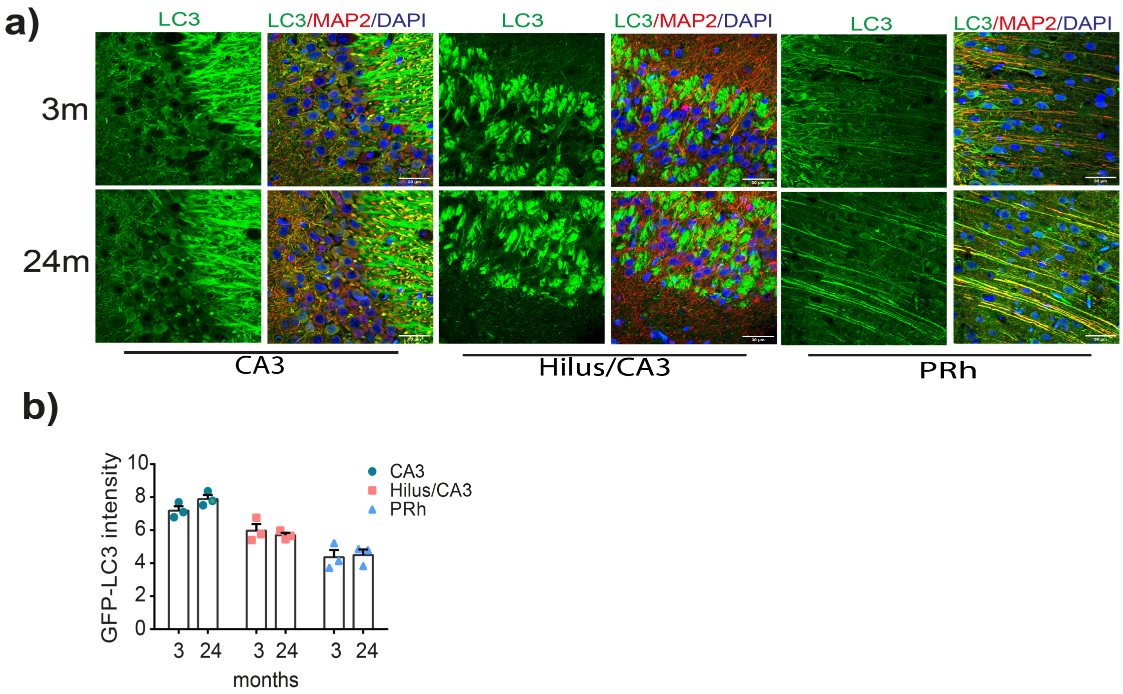

Supplementary Figure S3. Pattern distribution and expression level of GFP-LC3 did not change during brain aging. a) Confocal images showing GFP-LC3 signal in neurons (expressing MAP2) of hippocampus and perirhinal cortex (PRh) from 3 and 24 months-old GFP-LC3 transgenic mice. **b)** Graph represents pixel density of GFP-LC3 signal in the different brain regions analyzed in three brains per age. We noticed an increment of GFP-LC3 along axons in PRh region, but quantifying GFP-LC3 intensity we found no significant difference. Bars represent the mean ± SEM**.**
